# Identification of a Fungi-Specific Lineage of Protein Kinases Closely Related to Tyrosine Kinases

**DOI:** 10.1371/journal.pone.0089813

**Published:** 2014-02-27

**Authors:** Zhongtao Zhao, Qiaojun Jin, Jin-Rong Xu, Huiquan Liu

**Affiliations:** 1 State Key Laboratory of Crop Stress Biology for Arid Areas, College of Plant Protection, Northwest A&F University, Yangling, Shaanxi, China; 2 Department of Botany and Plant Pathology, Purdue University, West Lafayette, Indiana, United States of America; University of California-Riverside, United States of America

## Abstract

Tyrosine kinases (TKs) specifically catalyze the phosphorylation of tyrosine residues in proteins and play essential roles in many cellular processes. Although TKs mainly exist in animals, recent studies revealed that some organisms outside the Opisthokont clade also contain TKs. The fungi, as the sister group to animals, are thought to lack TKs. To better understand the origin and evolution of TKs, it is important to investigate if fungi have TK or TK-related genes. We therefore systematically identified possible TKs across the fungal kingdom by using the profile hidden Markov Models searches and phylogenetic analyses. Our results confirmed that fungi lack the orthologs of animal TKs. We identified a fungi-specific lineage of protein kinases (FslK) that appears to be a sister group closely related to TKs. Sequence analysis revealed that members of the FslK clade contain all the conserved protein kinase sub-domains and thus are likely enzymatically active. However, they lack key amino acid residues that determine TK-specific activities, indicating that they are not true TKs. Phylogenetic analysis indicated that the last common ancestor of fungi may have possessed numerous members of FslK. The ancestral FslK genes were lost in *Ascomycota* and *Ustilaginomycotina* and *Pucciniomycotina* of *Basidiomycota* during evolution. Most of these ancestral genes, however, were retained and expanded in *Agaricomycetes*. The discovery of the fungi-specific lineage of protein kinases closely related to TKs helps shed light on the origin and evolution of TKs and also has potential implications for the importance of these kinases in mushroom fungi.

## Background

Proteins undergo various post-translational modifications such as ribosylation, acetylation, thiolation, and phosphorylation. In eukaryotic organisms, reversible protein phosphorylation achieved by protein kinases (PKs) and phosphatases plays critical roles in the regulation of enzyme activity and intracellular signaling. Most PKs catalyze ATP-dependent phosphorylation of Serine (Ser) or Threonine (Thr), and some of these, which are known as dual-specificity kinases, can also phosphorylate on tyrosine (Tyr) [1], [2], [3]. Tyrosine kinase (TK) is a distinct group that specially catalyzes the phosphorylation of Tyr residues in proteins. In animals, TKs play essential roles in cell proliferation and differentiation, immune responses, organ development, and other cellular processes [4], [5]. Mutations in TK genes have been linked to various human diseases, such as cancer and immune diseases [6], [7], [8].

The Ser/Thr kinase catalytic domains are highly conserved and could be divided into 11 subdomains [1]. Tyrosine kinases contain highly conserved catalytic domains similar to those in protein Ser/Thr kinases but with unique subdomain motifs. Three motifs in subdomain VI, VIII and XI are highly conserved in TKs but are not found in Ser/Thr kinase [1], [9], [10]. The high degree of conservation of the tyrosine kinase motifs could be used to distinguish TKs from Ser/Thr kinases.

To date, most TKs were found in metazoan species. Previous studies have demonstrated that TK genes underwent duplication and loss during the evolution of metazoans [11]. A further study on dozens of eukaryotic genomes revealed that TKs appeared early in the common ancestor of metazoans and expanded after the divergence of the metazoans, especially after the split of the vertebrate lineage from the Ciona linage [12]. More recently, TKs were demonstrated to be established before the divergence of filastereans from the Metazoa and Choanoflagellata clades [13]. Many organisms outside the Opisthokont clade, such as Amoebozoa *Acanthamoeba castellanii*, *Dictyostelium discoideum* and *Entamoeba histolytica* [13], green alga *Chlamydomonas reinhardtii* [14], and oomycete *Phytophthora infestans* [15] were also found to contain TK or putative TK genes.

Fungi were found to have tyrosine kinase-like kinases (TKLs) [16], a group of kinases that share high sequence similarity with TKs but function mainly as serine-threonine kinases. However, it is generally thought that fungi lack TKs [13], [16], [17]. Recently, possible TK genes were identified in the basidiomycete *Laccaria bicolor* using sequence searches [16] but whether these genes are true TKs remains to be determined. To systematically investigate whether TK genes occurred in fungi, in this study we searched for possible TKs across the fungal kingdom by using Profile hidden Markov models (HMMs) [17] and determined their relationships with TKs by phylogenetic analysis. Our results confirmed that fungi lack orthologs of animal TKs. However, they have a specific lineage of protein kinases which is most closely related to TKs. Most of these genes were found in *Agaricomycetes* of *Basidiomycota* but neither in *Ascomycota* nor other phyla of *Basidiomycota*. Members of this lineage are predicted to have enzymatic activity but lack key amino acid residues that determine TK-specific activity. The evolution of members of this lineage was also addressed.

## Results and Discussion

### Identification of possible TKs in fungi

**Figure 1:**
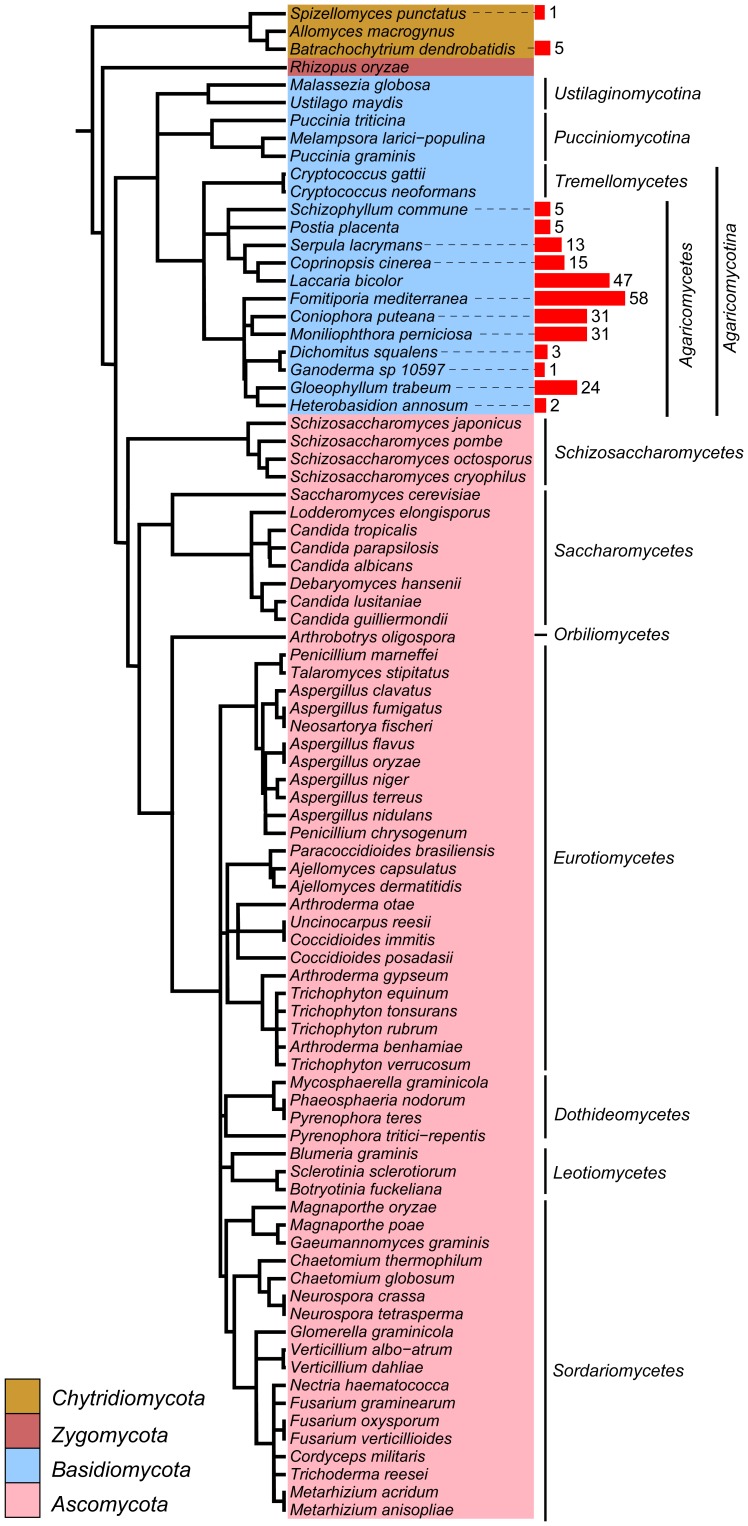
The distribution of FslK members in fungal species used in this study. The species tree was drawn based on the phylogenetic tree of α-tubulins. The red bar indicates the number of FslK members.

To systematically search for the fungal possible TKs, we used the HMMER program [18] to search the predicted proteomes of 84 fungi from phyla *Ascomycota*, *Basidiomycota*, *Chytridiomycota*, and *Zygomycota* (Figure 1; Table S1) with the multi-level HMM library of protein kinases [17]. Only the fungal sequences designated as TKs (best matches) were selected and then deposited into the Pfam server for kinase domain confirmation. These sequences were further subject to preliminary phylogenetic analysis with classical TKs and TKLs downloaded from Kinbase (http://kinase.com/kinbase/). We also included some representative fungal sequences classified as TKLs by our HMMER searches. In the resulting phylogenetic tree, fungal sequences identified as TKs were clustered into two distinct clades (Figure S1). One clade (fungal clade 2) was clustered into TKLs and was thus excluded from the following analysis. The other clade (fungal clade 1) was most closely related to animal TKs. To identify new sequences belonging to this clade, we built a HMM profile with the 18 sequences of fungal clade 1 and combined it into the kinase HMM library to further search against fungal proteomes. The close relationships of newly identified sequences with TKs were also confirmed by the phylogenetic analysis. In total, we identified 241 sequences from 14 fungi (Figure 1; Table S2). These sequences formed a distinct clade in the phylogenetic tree. We named this clade as fungi-specific lineage of protein kinase (FslK).

### Phylogenetic position of the FslK

Because organisms beyond animals, including Amoebozoa *A. castellanii*, *D. discoideum* and *E. histolytica*, and Oomycete *P. infestans* were also found to contain TKs. We therefore performed a comprehensive phylogenetic analysis with selected representative members of FslK to determine their evolutionary relationship with all known TKs, by using two independent phylogenetic methodologies: Maximum likelihood (ML) and Bayesian inference (BI). The green alga *C. reinhardtii* was also reported to have TKs [14]. We identified 16 possible TK sequences from *C. reinhardtii* genome and included them in our analysis.

**Figure 2:**
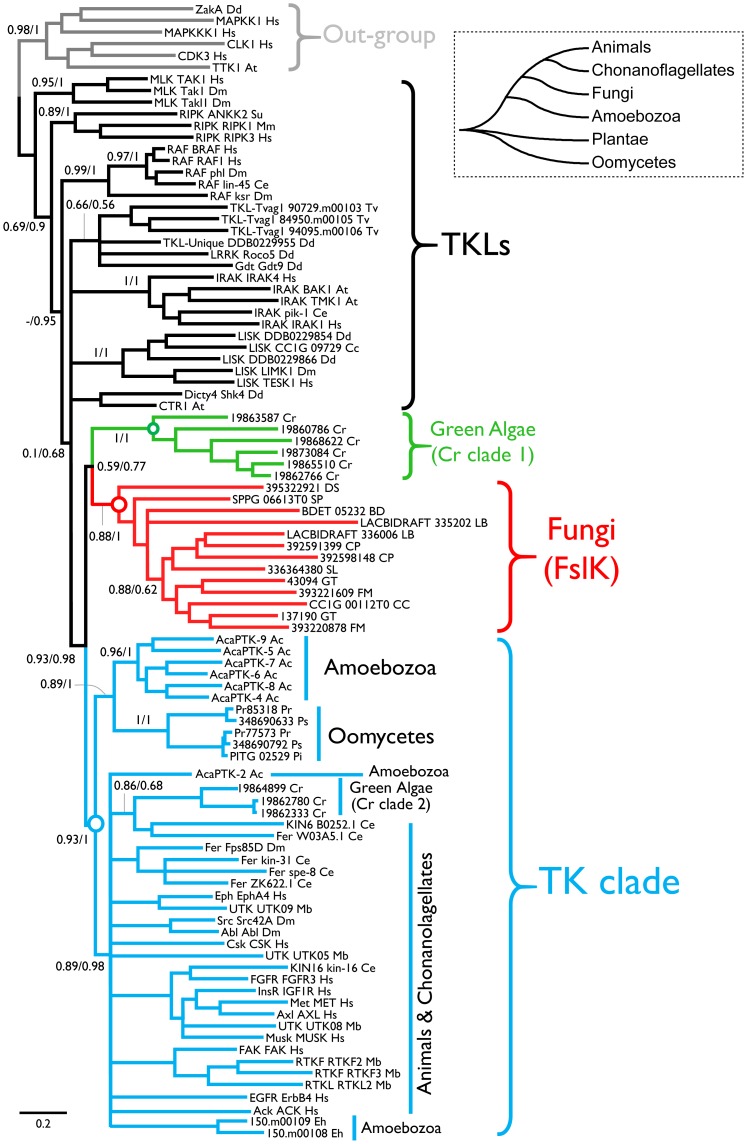
Phylogenetic position of the FslK. Phylogenetic trees were calculated using Maximum-likelihood (ML) and Bayesian inference (BI) methods, respectively. Both methodologies gave similar tree topology. The tree presented here is the BI tree. Numbers on major branches indicate SH-like approximate likelihood ratio test (SH-aLRT) probabilities/Bayesian posterior probabilities. Branches with Bayesian posterior probability less than 0.5 have been collapsed. The simple cladogram of eukaryotic groups on the top right corner was drawn according to the tree of life (http://tolweb.org/tree/). Ac, *Acanthamoeba castellanii*; At, *Arabidopsis thaliana*; Ce, *Caenorhabditis elegans*; Cr, *Chlamydomonas reinhardtii*; Dd, *Dictyostelium discoideum*; Dm, *Drosophila melanogaster*; Eh, *Entamoeba histolytica*; Hs, *Homo sapiens*; Mb, *Monosiga brevicollis*; Mm, *Mus musculus*; Pi, *Phytophthora infestans*; Pr, *Phytophthora ramorum*; Ps, *Phytophthora sojae*; Su, *Sea Urchin*; Tv, *Trichomonas vaginalis*. For abbreviations of fungi see Table S1.

In the resulting ML and BI trees (Figure 2), the known TKs, including classic TKs from animals and choanoflagellates, and previously reported TKs from pre-opisthokont species *E. histolytica*, *A. castellanii*, *P. infestans*, formed a well-supported clade (named as TK clade). Three sequences of *C. reinhardtii* also fell into the TK clade. The FslK was clustered with a clade of *C. reinhardtii* (Cr clade 1) and together formed a sister group to the TK clade. As we know, fungi are evolutionary more close to animals than those of pre-opisthokont species. If fungi have orthologs of animal TKs, they should be clustered with them in the TK clade. In contrast, the position of the FslK clade suggests that orthologs of animal TKs were lost in fungi.

Since the TK activity of members in Cr clade 1 is unclear, we do not know if the last common ancestor of both TK clade and Cr clade 1 has the TK activities. Therefore, whether the FslK members have TK activity cannot be determined solely by the phylogenetic position.

### The members of FslK may have no TK activity

**Figure 3:**
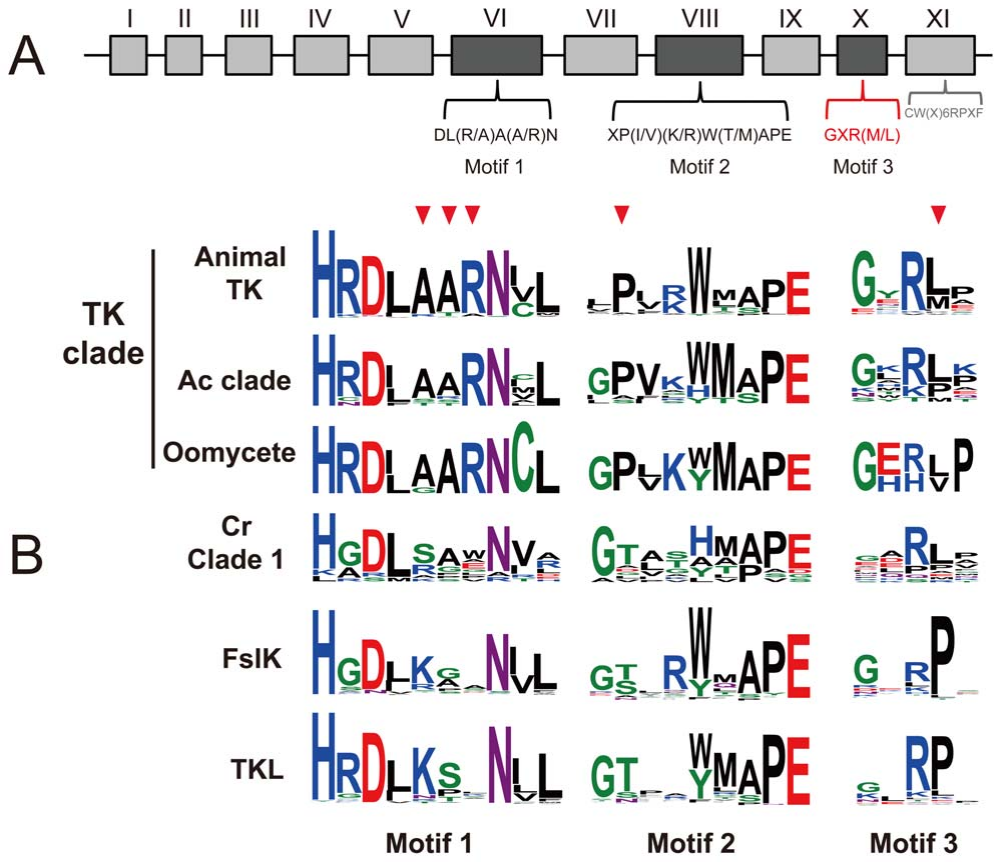
Comparison of sequence patterns in TK-specific motifs. **A**. Sub-domains of the protein kinase domain. Consensus sequences DL(R/A)A(A/R)N in subdomain VI and XP(I/V)(K/R)W(T/M)APE in subdomain VIII are specific to TKs. The motif in red [GXR(M/L)] was identified in this study. The motif CW(X)_6_RPXF in gray was found to be not specific to TKs in this study (Figure S2). **B**. The LOGOs show sequence patterns of the three motifs in each group. Red arrow heads indicate conserved amino acid residues that are diagnostic for TKs.

We performed comparative analysis of TK unique motifs and specific residues related to TK activities in catalytic domain to explore whether FslK members have tyrosine catalytic activities. The three motifs in subdomain VI, VIII and XI are reported to be TK specific [1], [9], [10]. However, in our analysis the sequence pattern of the motif in subdomain X [CW(X)_6_RPXF] was found to be shared by TKs and TKLs (Figure S2) and therefore was excluded from our subsequent analysis. A new motif with sequence pattern [GXR(L/M)] in subdomain X was found to be TK specific (Figure S2) and was used in our analysis. Results of comparative analysis showed that the sequence patterns of FslK are obviously different from those of TKs (Figure 3). The key amino acid residues ‘AARN’ for stabilizing the relative positions of the substrate-binding site and the catalytic loop of TKs in motif 1 and the first conserved proline (P) residue important for substrate recognition in motif 2 [10], [19] were not found in FslK members. In addition, TKs have a glutamate (L) or methionine (M) residue in the fourth position of motif 3, while members of FslK have a ‘P’ residue in the equivalent site. These residues are important for the TK activity and are diagnostic for TKs, and the lacking of these residues in the members of FslK suggests that they have no TK activities. Members of Cr clade 1 do not contain these key residues, and thus may also have no TK activities.

TKs, especially metazoan TKs, contain additional domains out of catalytic domains [12]. We examined additional domains in the FslK members. Different from the TKs, most of the FslK members have no additional domains; only 16 of the 241 members (Table S3) have additional domains which are not likely related to TK activities.

These results together with the phylogenetic analysis suggest that the TK activity is most likely to be acquired by the ancestor of TK clade after it diverged from the last common ancestor of FslK and Cr clade 1.

### Distribution and evolution of FslK members

Among the 241 FslK members, only 6 are from *Chytridiomycota*. All the others are from *Agaricomycotina* of *Basidiomycota*. Surprisingly, no FslK sequences were detected in 62 ascomycetes examined. In *Agaricomycotina*, only the mushroom-forming fungi *Agaricomycetes* contain FslK members but the two Tremellomycetes, *Cryptococcus neoformans* and *Cryptococcus gattii*, lack any putative FslK (Figure 1).

**Figure 4:**
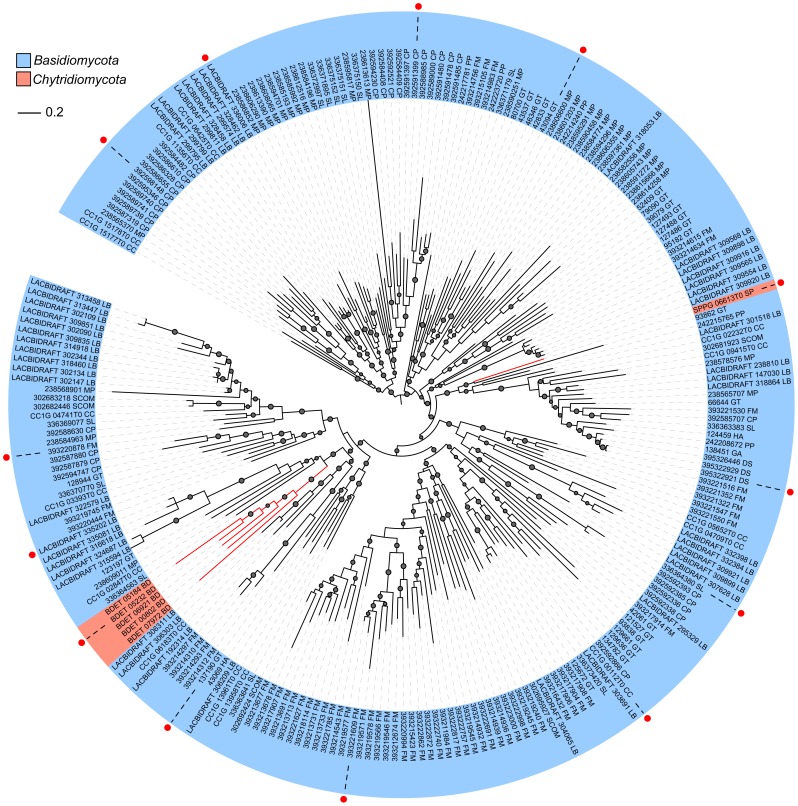
The maximum likelihood tree of FslK members. The phylogenetic tree was built with the kinase domain sequences using PhyML 3.1 and was drawn using Interactive Tree Of Life Version 2.2.2 (http://itol.embl.de/#). The p-values of approximate likelihood ratios (SH-aLRT) plotted as circle marks on the branches (only p-values>0.5 are indicated) and circle size is proportional to the p-values. Filled red circles mark sequences used in the Figure 2. For abbreviations see Table S1.

Phylogenetic analysis showed that FslK members were clustered into multiple sub-clades. Each sub-clade contains sequences from different species. Moreover, two distantly related sub-clades both contain sequences from Basidiomycetes and Chytridiomycetes (Figure 4). These suggest that the last common ancestor of fungi had possessed numerous paralogous genes from which the sub-clades were descended. All these ancestral genes may have been lost in Ascomycetes and also in *Ustilaginomycotina* and *Pucciniomycotina* of *Basidiomycota*. In contrast, *Agaricomycetes* retained most of these copies. Furthermore, lineage or species-specific gene duplications (gains) also have occurred in some *Agaricomycetes*. For example, the wood decaying fungus *Fomitiporia mediterranea* and the ectomycorrhizal basidiomycete *L. bicolor* each contains numerous FslK members, and many of them in each species have high sequence identity and are clustered together in the phylogenetic tree (Figure 4), suggesting recent expansion occurred in these species.

### Members of FslK may have important functions in *Agaricomycetes*

**Figure 5:**
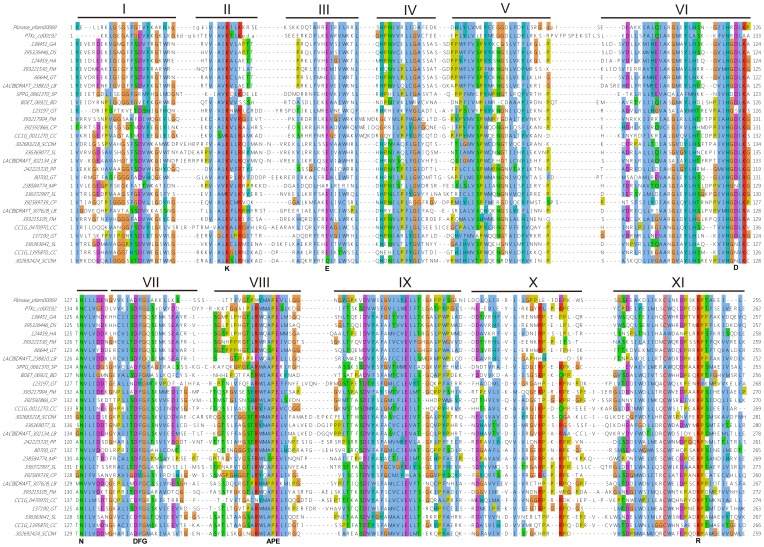
Multiple sequence alignments of representative members of FslK. The consensus sequences of Protein kinase domain (Pkinase, pfam00069) and Catalytic domain of Protein Tyrosine Kinases (PTKc, cd00192) were used as references. Eleven sub-domains of FslK catalytic domains were shown. Conserved amino acid residues related to crystal structure and catalytic function [1], [9], [10] in protein kinases were indicated below. The default color scheme for ClustalW alignment in the Jalview program was used.

Sequence conservation of proteins is correlated with their functions, and proteins with important molecular functions are more conserved because they are under higher selection pressure than those of less important ones [20], [21]. Alignment of FslK sequences revealed that although some of them are truncated in the kinase catalytic domains, most of them have complete catalytic domains, and are highly conserved in residues required for catalysis, such as residues required for ATP and substrate binding (Figure 5). This suggests that members of FslK have catalytic activities of protein kinases. Carefully examined the sequences, we found many of the FslK members with incomplete catalytic domains are truncated due to sequencing gaps or wrong annotation, and the truncation of the others may be due to the sequence degradation caused by functional redundancy after recent gene duplication.

We further investigated the expression of FslK genes by searching for corresponding EST data from NCBI and JGI database. Most genes examined were found to be expressed (Table S4), suggesting that these genes are functional in these organisms.

Taken together, above evidences suggest that the FslK genes likely play important roles in fungi. Considering that multiple ancestral genes of FslK members were retained and further expanded in *Agaricomycetes*, some members of FslK most likely play important roles in controlling cellular development and differentiation processes specific to mushroom-forming fungi. However, their exact functions need to be experimentally determined.

In summary, we systematically investigated possible TKs in fungi by using HMMs and phylogenetic analysis. Our results confirmed that fungi lack the orthologs of animal TKs. However, there is a specific lineage of protein kinases in fungi (FslK) which is most closely related to TKs. Kinases of the FslK clade lack key amino acid residues that determine TK-specific activities and therefore may not be true TKs. However, they contain conserved catalytic domains of protein kinases and thus are likely enzymatically active. Phylogenetic analysis revealed that the last common ancestor of fungi had possessed several FslK genes. The ancestral FslK genes may have been lost in *Ascomycota* and also in *Ustilaginomycotina* and *Pucciniomycotina* of *Basidiomycota* during evolution. However, most of these ancestral genes were retained and further expanded in *Agaricomycetes*, suggesting that the FslK kinases possibly have important functions in controlling cellular processes specific to mushroom fungi. This discovery of the FslK protein kinases closely related to TKs helps shed light on the origin and evolution of TKs and also has potential implications for the importance of these kinases in mushroom fungi.

## Materials and Methods

### Data collection

The predicted proteomes (listed in Table S1) of fungal genomes used in this study were downloaded from the Fungal Genome Initiative (FGI) site at the Broad Institute (http://www.broadinstitute.org), GenBank of NCBI, DOE Joint Genome Institute (JGI) (http://genome.jgi.doe.gov/programs/fungi/index.jsf), and BluGen (http://www.blugen.org/). The predicted proteome of *C. reinhardtii* was downloaded from the JGI phytozome site (http://www.phytozome.net/) [22]. The HMM Library with all protein kinase domain definitions was downloaded from the Kinomer database (http://www.compbio.dundee.ac.uk/kinomer/index.html) [17].

Catalytic domain sequences of TKs and TKLs from Homo sapiens, Drosophila melanogaster, Caenorhabditis elegans, M. brevicollis, Trichomonas vaginalis, Tetrahymena thermophila, and TKLs of Coprinopsis cinerea were obtained from the Kinbase (the kinase database, http://kinase.com/kinbase) that collected the currently accepted classification of eukaryotic kinases [23]. The catalytic domains of putative TKs of Entamoeba histolytica were obtained from the Kinomer database. TKs of A. castellanii and three Oomycetes P. infestans, P. sojae, and P. ramorum identified in previous studies [13], [15] were retrieved from GenBank.

### Identification of possible TKs in fungi

The Hmmscan program in the HMMER 3.0 package [18] was employed to search the multi-level HMM library of protein kinases with each fungal proteome as queries using score of 20 as the cutoff. Only the fungal sequences designated as TKs (best matches) were selected and deposited into the Pfam server to confirm if they are kinases. These sequences were further subject to the preliminary phylogenetic analysis with classic TKs and TKLs to determine if they are closely related to TKs.

### Sequence alignment and phylogenetic analysis

Multiple sequence alignments were performed with the PSI-Coffee program [24]. Alignments used for phylogenetic analysis were trimmed by trimAL [25] with gappyout model. Some sequences that are truncated due to wrong gene prediction were manually revised.

Phylogenetic trees were constructed with two independent methods: Maximum likelihood (ML) and Bayesian inference (BI) methodologies. The ML trees were constructed with PhyML 3.1 [26] using the best-fit model LG+Γ selected by ProtTest3 [27], with SPRs algorithms and 16 categories of γ-distributed substitution rates. The reliability of internal branches was evaluated with SH-aLRT supports. The BI tree was constructed with MrBayes-3.2 [28] using mixed models of amino acid substitution with 16 categories of γ-distributed substitution rates, performing two runs for each of four Monte Carlo Markov Chains (MCMCs), sampling every 1000th iteration over 1.1×10^6^ generations after a burn-in of 101 samples.

### Examination of functional domains

Conserved protein domains were searched in the Pfam database [29]at Sanger and the CDD database at NCBI (http://www.ncbi.nlm.nih.gov/cdd). Sequence logos were generated by WebLogo (http://weblogo.berkeley.edu/) [30].

## Supporting Information

Figure S1 **Phylogenetic analysis of fungal sequences classified as TKs with those of classical TKs and TKLs.** The phylogenetic tree was built with the kinase domain sequences using ML methodologies with SPRs algorithms and 16 categories of γ-distributed substitution rates. The reliability of internal branches was evaluated based on SH-aLRT supports. The base tree was drawn using Interactive Tree Of Life Version 2.2.2 (http://itol.embl.de/#). The p-values of approximate likelihood ratios (SH-aLRT) are plotted as circle marks on the branches (only p-values>0.5 are indicated) and circle size is proportional to the p-values. Sequences in fungal clade 1 and clade 2 were designated as TKs by multi-level HMM library of protein kinases. Other fungal sequences were designated as TKLs. Abbreviated species names are as follows: At, *Arabidopsis thaliana*; Ce, *Caenorhabditis elegans*; Cr, *Chlamydomonas reinhardtii*; Dd, *Dictyostelium discoideum*; Dm, *Drosophila melanogaster*; Eh, *Entamoeba histolytica*; Hs, *Homo sapiens*; Mb, *Monosiga brevicollis*; Mm, *Mus musculus*; Ot, *Ostreococcus tauri*; Ol, *Ostreococcus lucimarinus*; Su, Sea Urchin; Tt, *Tetrahymena thermophila*; Tv, *Trichomonas vaginalis*; Vv, *Vitis vinifera*. For abbreviations of fungi see Table S1.
(PDF)

Figure S2 **The identification of a new TK-specific motif in sub-domain X.** Asterisks indicate the newly identified TK-specific motif [GXR(M/L)]. The previous reported motif [CW(X)_6_RPXF] shaded in gray is common in TKs and TKLs.
(PDF)

Table S1 **Information of fungal species used in this study.**
(XLSX)

Table S2 **Catalytic domains of FslK kinases.**
(XLSX)

Table S3 **Additional domains of FslK kinases.**
(XLSX)

Table S4 **FslK genes detected to be expressed.**
(XLSX)

## References

[1] Hanks SK, Hunter T (1995) Protein kinases 6. The eukaryotic protein kinase superfamily: kinase (catalytic) domain structure and classification. FASEB J 9: 576–596. 7768349

[2] Rudrabhatla P, Reddy MM, Rajasekharan R (2006) Genome-wide analysis and experimentation of plant serine/ threonine/tyrosine-specific protein kinases. Plant Mol Biol 60: 293–319. 16429265 10.1007/s11103-005-4109-7

[3] Dhanasekaran N, Premkumar Reddy E (1998) Signaling by dual specificity kinases. Oncogene 17: 1447–1455. 9779990 10.1038/sj.onc.1202251

[4] Hubbard SR, Till JH (2000) Protein tyrosine kinase structure and function. Annu Rev Biochem 69: 373–398. 10966463 10.1146/annurev.biochem.69.1.373

[5] Hunter T (1998) The Croonian Lecture 1997. The phosphorylation of proteins on tyrosine: its role in cell growth and disease. Philos Trans R Soc Lond B Biol Sci 353: 583–605. 9602534 10.1098/rstb.1998.0228 PMC1692245

[6] Blume-Jensen P, Hunter T (2001) Oncogenic kinase signalling. Nature 411: 355–365. 11357143 10.1038/35077225

[7] Mustelin T, Feng GS, Bottini N, Alonso A, Kholod N, et al (2002) Protein tyrosine phosphatases. Front Biosci 7: d85–142. 11779706 10.2741/A770

[8] Alonso A, Sasin J, Bottini N, Friedberg I, Osterman A, et al (2004) Protein tyrosine phosphatases in the human genome. Cell 117: 699–711. 15186772 10.1016/j.cell.2004.05.018

[9] Taylor SS, Radzio-Andzelm E, Hunter T (1995) How do protein kinases discriminate between serine/threonine and tyrosine? Structural insights from the insulin receptor protein-tyrosine kinase. FASEB J 9: 1255–1266. 7557015 10.1096/fasebj.9.13.7557015

[10] Hanks SK (2003) Genomic analysis of the eukaryotic protein kinase superfamily: a perspective. Genome Biol 4: 111. 12734000 10.1186/gb-2003-4-5-111 PMC156577

[11] Popovici C, Roubin R, Coulier F, Pontarotti P, Birnbaum D (1999) The family of *Caenorhabditis elegans* tyrosine kinase receptors: similarities and differences with mammalian receptors. Genome Res 9: 1026–1039. 10568743 10.1101/gr.9.11.1026

[12] Shiu SH, Li WH (2004) Origins, lineage-specific expansions, and multiple losses of tyrosine kinases in eukaryotes. Mol Biol Evol 21: 828–840. 14963097 10.1093/molbev/msh077

[13] Suga H, Dacre M, de Mendoza A, Shalchian-Tabrizi K, Manning G, et al (2012) Genomic survey of premetazoans shows deep conservation of cytoplasmic tyrosine kinases and multiple radiations of receptor tyrosine kinases. Sci Signal 5: ra35. 22550341 10.1126/scisignal.2002733

[14] Wheeler GL, Miranda-Saavedra D, Barton GJ (2008) Genome analysis of the unicellular green alga *Chlamydomonas reinhardtii* Indicates an ancient evolutionary origin for key pattern recognition and cell-signaling protein families. Genetics 179: 193–197. 18493051 10.1534/genetics.107.085936 PMC2390598

[15] Judelson HS, Ah-Fong AM (2010) The kinome of *Phytophthora infestans* reveals oomycete-specific innovations and links to other taxonomic groups. BMC Genomics 11: 700. 21143935 10.1186/1471-2164-11-700 PMC3019232

[16] Kosti I, Mandel-Gutfreund Y, Glaser F, Horwitz BA (2010) Comparative analysis of fungal protein kinases and associated domains. BMC Genomics 11: 133. 20178650 10.1186/1471-2164-11-133 PMC2838846

[17] Miranda-Saavedra D, Barton GJ (2007) Classification and functional annotation of eukaryotic protein kinases. Proteins 68: 893–914. 17557329 10.1002/prot.21444

[18] Eddy SR (2011) Accelerated Profile HMM Searches. PLoS Comput Biol 7: e1002195. 22039361 10.1371/journal.pcbi.1002195 PMC3197634

[19] Cowan-Jacob SW (2006) Structural biology of protein tyrosine kinases. Cell Mol Life Sci 63: 2608–2625. 17041812 10.1007/s00018-006-6202-8 PMC11136174

[20] Carlson MR, Zhang B, Fang Z, Mischel PS, Horvath S, et al (2006) Gene connectivity, function, and sequence conservation: predictions from modular yeast co-expression networks. BMC Genomics 7: 40. 16515682 10.1186/1471-2164-7-40 PMC1413526

[21] Cooper GM, Brown CD (2008) Qualifying the relationship between sequence conservation and molecular function. Genome Res 18: 201–205. 18245453 10.1101/gr.7205808

[22] Merchant SS, Prochnik SE, Vallon O, Harris EH, Karpowicz SJ, et al (2007) The Chlamydomonas genome reveals the evolution of key animal and plant functions. Science 318: 245–250. 17932292 10.1126/science.1143609 PMC2875087

[23] Manning G, Plowman GD, Hunter T, Sudarsanam S (2002) Evolution of protein kinase signaling from yeast to man. Trends Biochem Sci 27: 514–520. 12368087 10.1016/s0968-0004(02)02179-5

[24] Di Tommaso P, Moretti S, Xenarios I, Orobitg M, Montanyola A, et al (2011) T-Coffee: a web server for the multiple sequence alignment of protein and RNA sequences using structural information and homology extension. Nucleic Acids Res 39: W13–17. 21558174 10.1093/nar/gkr245 PMC3125728

[25] Capella-Gutierrez S, Silla-Martinez JM, Gabaldon T (2009) trimAl: a tool for automated alignment trimming in large-scale phylogenetic analyses. Bioinformatics 25: 1972–1973. 19505945 10.1093/bioinformatics/btp348 PMC2712344

[26] Guindon S, Dufayard JF, Lefort V, Anisimova M, Hordijk W, et al (2010) New algorithms and methods to estimate maximum-likelihood phylogenies: assessing the performance of PhyML 3.0. Syst Biol 59: 307–321. 20525638 10.1093/sysbio/syq010

[27] Darriba D, Taboada GL, Doallo R, Posada D (2011) ProtTest 3: fast selection of best-fit models of protein evolution. Bioinformatics 27: 1164–1165. 21335321 10.1093/bioinformatics/btr088 PMC5215816

[28] Ronquist F, Teslenko M, van der Mark P, Ayres DL, Darling A, et al (2012) MrBayes 3.2: efficient Bayesian phylogenetic inference and model choice across a large model space. Syst Biol 61: 539–542. 22357727 10.1093/sysbio/sys029 PMC3329765

[29] Punta M, Coggill PC, Eberhardt RY, Mistry J, Tate J, et al (2012) The Pfam protein families database. Nucleic Acids Res 40: D290–301. 22127870 10.1093/nar/gkr1065 PMC3245129

[30] Crooks GE, Hon G, Chandonia JM, Brenner SE (2004) WebLogo: a sequence logo generator. Genome Res 14: 1188–1190. 15173120 10.1101/gr.849004 PMC419797

