# Supplementary figures and images for: Identification of a Fungi-Specific Lineage of Protein Kinases Closely Related to Tyrosine Kinases
Source: PLoS One. 2014 Feb 27;9(2):e89813. doi: 10.1371/journal.pone.0089813 (PMC3937382; doi:10.1371/journal.pone.0089813)

- Animals
- Choanoflagellates
- Amoebozoa
- Plants
- Fungi
- Alveolates
- Green algae
- Parabasalids

0.5

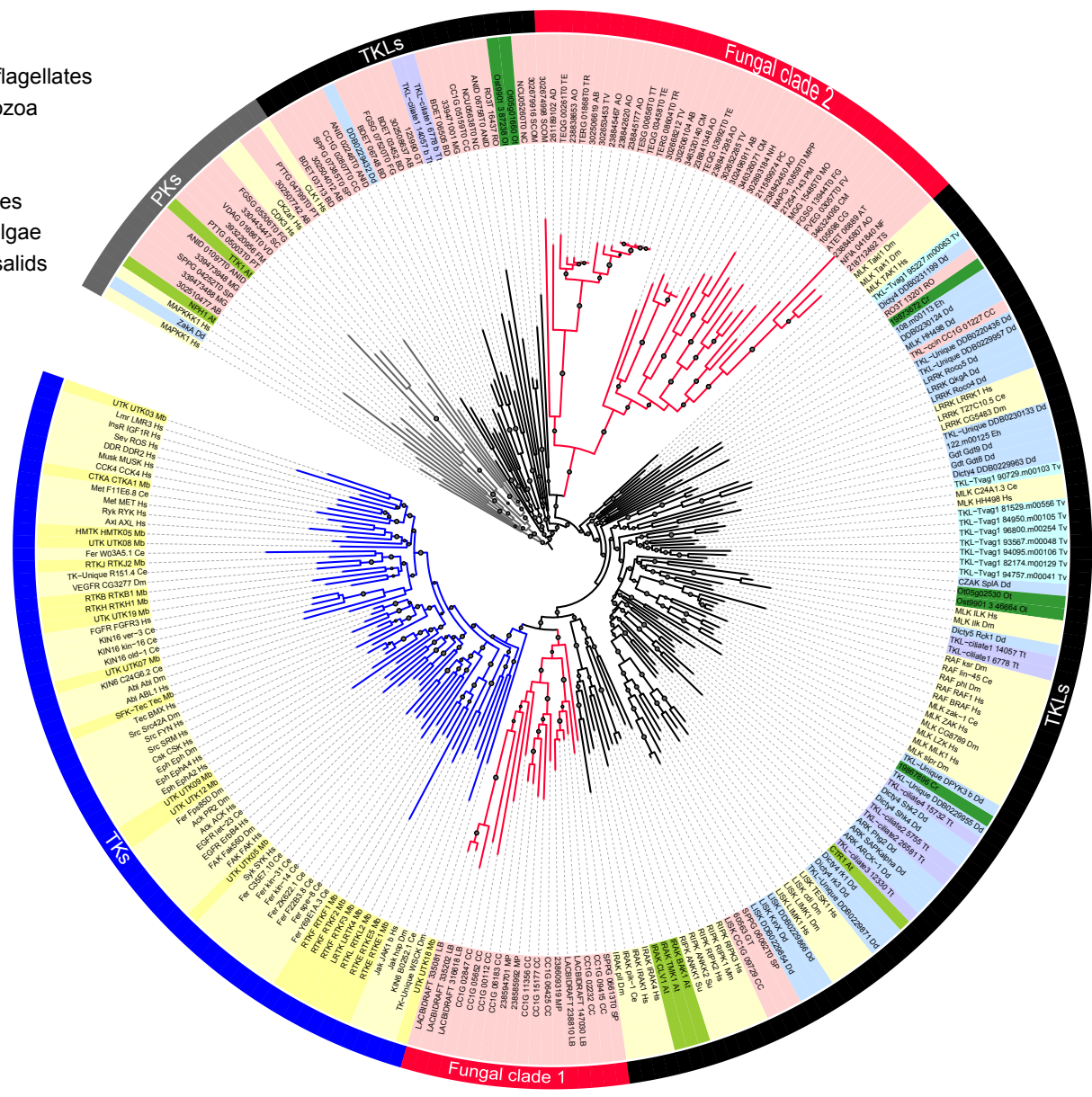

Supplement: Figure S1 — Phylogenetic analysis of fungal sequences classified as TKs with those of classical TKs and TKLs. The phylogenetic tree was built with the kinase domain sequences using ML methodologies with SPRs algorithms and 16 categories of γ-distributed substitution rates. The reliability of internal branches was evaluated based on SH-aLRT supports. The base tree was drawn using Interactive Tree Of Life Version 2.2.2 (http://itol.embl.de/#). The p-values of approximate likelihood ratios (SH-aLRT) are plotted as circle marks on the branches (only p-values>0.5 are indicated) and circle size is proportional to the p-values. Sequences in fungal clade 1 and clade 2 were designated as TKs by multi-level HMM library of protein kinases. Other fungal sequences were designated as TKLs. Abbreviated species names are as follows: At, Arabidopsis thaliana; Ce, Caenorhabditis elegans; Cr, Chlamydomonas reinhardtii; Dd, Dictyostelium discoideum; Dm, Drosophila melanogaster; Eh, Entamoeba histolytica; Hs, Homo sapiens; Mb, Monosiga brevicollis; Mm, Mus musculus; Ot, Ostreococcus tauri; Ol, Ostreococcus lucimarinus; Su, Sea Urchin; Tt, Tetrahymena thermophila; Tv, Trichomonas vaginalis; Vv, Vitis vinifera. For abbreviations of fungi see Table S1. (PDF) [file pone.0089813.s001.pdf]
