# Supplementary material for: Identification of a Fungi-Specific Lineage of Protein Kinases Closely Related to Tyrosine Kinases
Source: PLoS One. 2014 Feb 27;9(2):e89813. doi: 10.1371/journal.pone.0089813 (PMC3937382; doi:10.1371/journal.pone.0089813)
Supplement: Figure S2 — The identification of a new TK-specific motif in sub-domain X. Asterisks indicate the newly identified TK-specific motif [GXR(M/L)]. The previous reported motif [CW(X)6RPXF] shaded in gray is common in TKs and TKLs. (PDF) [file pone.0089813.s002.pdf]

```
TKs
                                         * **
TK_VEGFR_CG3277_Dm          DELIPFL-MA-GNRLERP---EICTPQVYTIMLQCWLEEPEERPTF
TK_UTK_UTK19_Mb             MEAVAAI-AA-GYRLPKP---DACPDAMYDIMMRCWMYVPANRPDF
TK_Tec_BMX_Hs               SQVVLKV-SQ-GHRLYRP---HLASDTIYQIMYSCWHELPEKRPTF
TK_Syk_SYK_Hs               SEVTAML-EK-GERMGCP---AGCPREMYDLMNLCWTYDVENRPGF
TK_Src_FYN_Hs               REVLEQV-ER-GYRMPCP---QDCPISLHELMIHCWKKDPEERPTF
TK_SFK-Tec_Tec_Mb           AETVDRV-GR-GERMGRP---SLASELIYDIMSMCWHQDPESRISF
TK_Sev_ROS_Hs               LDVLNYV-QT-GGRLEPP---RNCPDDLWNLMTQCWAQEPDQRPTF
TK_Ryk_RYK_Hs               FEMAAYL-KD-GYRIAQP---INCPDELFAVMACCWALDPEERPKF
TK_RTKB_RTKB1_Mb            GDVFNLL-EK-GYRLPTP---RQAPAEADAIARMCWSLEAEDRPTF
TK_Met_MET_Hs               FDITVYL-LQ-GRRLLQP---EYCPDPLYEVMLKCWHPKAEMRPSF
TK_Jak_hop_Dm               EDFLNRL-QS-GERLNRP---ASCPDFIYDLMQLCWHATPRSRPSF
TK_InsR_IGF1R_Hs            EQVLRFV-ME-GGLLDKP---DNCPDMLFELMRMCWQYNPKMRPSF
TK_HMTK_HMTK05_Mb           IEVQKRV-RE-GLRLTAP---EGCDPEFFELMCSCWERQPSQRPTF
TK_FGFR_FGFR3_Hs            EELFKLL-KE-GHRMDKP---ANCTHDLYMIMRECWHAAPSQRPTF
TK_Fer_Fps85D_Dm            SRARERI-DT-GYRMPTP---KSTPEEMYRLMLQCWAADAESRPHF
TK_FAK_FAK_Hs               NDVIGRI-EN-GERLPMP---PNCPPTLYSLMTKCWAYDPSRRPRF
TK_Eph_Eph_Dm               QDVIKSI-EK-GYRLPAP---MDCPEALYQLMLDCWQKQRTHRPTF
TK_EGFR_ErbB4_Hs            REIPDLL-EK-GERLPQP---PICTIDVYMVMVKCWMIDADSRPKF
TK_Csk_CSK_Hs               KDVVPRV-EK-GYKMDAP---DGCPPAVYEVMKNCWHLDAAMRPSF
TK_Axl_AXL_Hs               SEIYDYL-RQ-GNRLKQP---ADCLDGLYALMSRCWELNPQDRPSF
TK_Ack_ACK_Hs               SQILHKI-DKEGERLPRP---EDCPQDIYNVMVQCWAHKPEDRPTF
TK_Abl_Abl_Dm               TDVYHKL-DK-GYRMERP---PGCPPEVYDLMRQCWQWDATDRPTF
TKLs
TKL_TKL-Unique_Dd           YLELARV-REEGLPPMIP---PEIEGDLRKIIEACFHRDHTKRPNF
TKL_TKL-Unique_Dd           VQLALAV-TTKSLRPPIP---NAWPYQLSHLIQACWHQDPLKRPSF
TKL_TKL-Tvag1_Tv            NDIFQQV-VQRDERPNFT---RMTPAPLQKLIKRCWDRDPEMRPSF
TKL_TKL-ciliate3_Tt         QQIYGTVGYDENYQVEIP---VRGIPRYLNLMKKCLRRNPQERPTF
TKL_RAF_lin-45_Ce           DQILFMV-GRGYLRPDRSKIRHDTPKSMLKLYDNCIMFDRNERPVF
TKL_Ot05g02530_Ot           LQAAVGV-VQRGLRPAIS---TSCNPKLAQVMQSCWLADATQRPGF
TKL_MLK_Tak1_Dm             YTIQWKI-YK-GERPPLL---TTCPKRIEDLMTACWKTVPEDRPSM
TKL_MLK_MLK1_Hs             LAVAYGV-AMNKLALPIP---STCPEPFAKLMEDCWNPDPHSRPSF
TKL_Gdt_Gdt8_Dd             NDIPNRV-QD-GFRPTAD--LDTIDDDIRKIIECCWIKEKSKRPSF
TKL_Dicty4_Shk2_Dd          PQLVEGV-VNKKNRPIIP---DYFPTRLKDLLARCWDHYPSRRPSF
TKL_Dicty4_rk1_Dd           FQVIFAV-GREGMRPPVP---QNGPPKYIQLLIDCLNENPSHRPTM
TKL_Dicty4_Dd               VELARSV-CDKKLKPKIS---SSVPNFISSLIKDCLHNSPKKRPTM
TKL_Dicty4_Dd               MNAGILV-ASKGLRPELP---DNCDPNWKKLVVWCWSEDPNKRPSF
TKL_CZAK_SplA_Dd            MKMAHLA-AYESYRPPIP---LTTSSKWKEILTQCWDSNPDSRPTF
TKL_AT1G64300.1_At          DKMAKNI-RT-GERPLFP---FPSPKYLVSLIKRCWHSEPSQRPTF
Ser/Thr kinases
T01H8.1a_Ce                 NDTMTQI-LK--AKLSMP---HFLTQEAQSLLRALFKRNSQNRLGA
SgK494_Hs                   VAMLASV-TH--SDSEIP---ASLNQGLSLLLHELLCQNPLHRLRY
SGK1_Hs                     AEMYDNI-LN--KPLQLK---PNITNSARHLLEGLLQKDRTKRLGA
RSK1_Hs                     KETMTLI-LK--AKLGMP---QFLSTEAQSLLRALFKRNPANRLGS
ROCK1_Hs                    VGTYSKI-MNHKNSLTFP-DDNDISKEAKNLICAFLT-DREVRLGR
RHOK_Hs                     KELKHRI-IS--EPVKYP---DKFSQASKDFCEALLEKDPEKRLGF
R04A9.5_Ce                  ADKDKSI-LK--GKVRLP---PKLSASGKDLIKRIIKRDPTLRITI
PRKX_Hs                     FGIYQKI-LA--GKIDFP---RHLDFHVKDLIKKLLVVDRTRRLGN
PKN1_Hs                     EEVFDSI-VN--DEVRYP---RFLSAEAIGIMRRLLRRNPERRLGS
PKG1_Hs                     MKTYNII-LRGIDMIEFP---KKIAKNAANLIKKLCRDNPSERLGN
PKCa_Hs                     DELFQSI-ME--HNVSYP---KSLSKEAVSVCKGLMTKHPAKRLGC
pkc-2_Ce                    DELFTAI-TE--HNVSYP---KSLSKEAVSLCKALLIKNPSKRLGC
PKACa_Hs                    IQIYEKI-VS--GKVRFP---SHFSSDLKDLLRNLLQVDLTKRFGN
PDK1_Hs                     YLIFQKI-IK--LEYDFP---EKFFPKARDLVEKLLVLDATKRLGC
pdk-1_Ce                    YHLLKRI-QE--LDFSFP---EGFPEEASEIIAKILVRDPSTRITS
p70S6K_Hs                   KKTIDKI-LK--CKLNLP---PYLTQEARDLLKKLLKRNAASRLGA
MSK1_Hs                     AEISRRI-LK--SEPPYP---QEMSALAKDLIQRLLMKDPKKRLGC
MRCKa_Hs                    VETYGKI-MNHKERFQFPAQVTDVSENAKDLIRRLIC-SREHRLGQ
MAST1_Hs                    EELFGQV-IS--DDILWPEGDEALPTEAQLLISSLLQTNPLVRLGA
kin-1_Ce                    IQIYEKI-VS--GKVKFP---SHFSNELKDLLKNLLQVDLTKRYGN
GPRK4_Hs                    EEVDQRI-KN--DTEEYS---EKFSEDAKSICRMLLTKNPSKRLGC
F46F6.2_Ce                  EEIFDSI-IS--EDVRYP---RYLSVESIAIMRRLLRKVPEKRLGY
egl-4_Ce                    MKTYTLI-LKGVDALEIP--NRRIGKTATALVKKLCRDNPGERLGS
DMPK1_Hs                    AETYGKI-VHYKEHLSLPLVDEGVPEEARDFIQRLLC-PPETRLGR
CRIK_Hs                     ARTFNNI-MNFQRFLKFP-DDPKVSSDFLDLIQSLLC-GQKERLKF
C09G4.2_Ce                  MVTYNAI-LKGLEKWAWP---RFVTKEAIDMMLSLCKYEPTERLGF
BARK1_Hs                    HEIDRMT-LT--MAVELP---DSFSPELRSLLEGLLQRDVNRRLGC
akt-2_Ce                    GKLFELI-TT--CDLKFP---NRLSPEAVTLLSGLLERVPAKRLGA
AKT1_Hs                     EKLFELI-LM--EEIRFP---RTLGPEAKSLLSGLLKKDPKQRLGG
akt-1_Ce                    NKLFELI-MA--GDLRFP---SKLSQEARTLLTGLLVKDPTQRLGG
                            _________________________  ______________________
                                        X                        XI
```
